# Preparation of Hybrid Magnetic Nanoparticles for Sensitive and Rapid Detection of Phorate Residue in Celery Using SERS Immunochromatography Assay

**DOI:** 10.3390/nano14121046

**Published:** 2024-06-18

**Authors:** Xiangyang Li, Hean Qian, Jin Tao, Mingshuo Cao, Meng Wang, Wenlei Zhai

**Affiliations:** 1 Beijing Laboratory of Food Quality and Safety, Beijing Key Laboratory of Detection and Control of Spoilage Organisms and Pesticide Residue in Agricultural Product, College of Food Science and Engineering, Beijing University of Agriculture, Beijing 102206, China; 20047801@bua.edu.cn (X.L.); 15210849723@163.com (H.Q.); 2 Institute of Quality Standard and Testing Technology, Beijing Academy of Agriculture and Forestry Science, Beijing 100097, China; taojing8585@163.com (J.T.); 17832071720@163.com (M.C.)

**Keywords:** surface-enhanced Raman spectroscopy, immunochromatography assay, rapid detection, organophosphorus pesticide, phorate, magnetic nanoparticles, immunochromatographic test strip

## Abstract

Extensive use of pesticides in agricultural production has been causing serious health threats to humans and animals. Among them, phorate is a highly toxic organophosphorus insecticide that has been widely used in planting. Due to its harmful effects on human and animal health, it has been restricted for use in many countries. Analytical methods for the rapid and sensitive detection of phorate residues in agricultural products are urgently needed. In this study, a new method was developed by combining surface-enhanced Raman spectroscopy (SERS) and immunochromatography assay (ICA). Hybrid magnetic Fe_3_O_4_@Au@DTNB-Ab nanoprobes were prepared by modifying and growing Au nanoseeds on an Fe_3_O_4_ core. SERS activity of the nanoprobe was optimized by adjusting the concentration of the Au precursor. A rapid and sensitive assay was established by replacing the traditional colloidal gold-based ICA with hybrid SERS nanoprobes for SERS-ICA. After optimizing parameters including coating antibody concentrations and the composition and pH of the buffer solution, the limit of detection (LOD) for phorate could reach 1 ng/mL, with a linear range of 5~100 ng/mL. This LOD is remarkably lower than the maximum residue limit in vegetables and fruits set by the Chinese government. The feasibility of this method was further examined by conducting a spiking test with celery as the real sample. The result demonstrated that this method could serve as a promising platform for rapid and sensitive detection of phorate in agricultural products.

## 1. Introduction

Extensive use of pesticides in agricultural production has been causing serious challenges for the health of humans and animals worldwide [1]. Among them, phorate is a highly toxic organophosphorus insecticide that is widely used in vegetable and fruit cultivation [2,3]. Overdose exposure to phorate can lead to acute poisoning effects such as headache, dizziness, nausea, and anorexia. In some serious cases, pulmonary edema, cerebral edema, coma and respiratory paralysis have been reported [4]. Long-term, low-level exposure to phorate can cause a reduction in blood cholinesterase activity as well as symptoms such as muscular fasciculation and hyperhidrosis [5]. Recent research revealed that phorate could cause hyperglycemia and reduce the abundance of intestinal microbiota in mice [6]. In some extreme cases, intentional suicides after phorate ingestion have been reported in the literature [7,8]. Due to these harmful effects to human and animal health, phorate has been strictly forbidden for use in vegetables cultivation and cereal planting in many countries [9]. Maximum residue levels (MRLs) have been set for daily inspection in order to protect consumers from the potential risks. In China, the MRL for phorate is set at 0.01 mg/kg in vegetables and fruits (according to GB 2763-2021 [10]). Considering the low level of MRL, the development of sensitive and reliable methods for the detection of phorate in agricultural products is urgently needed.

Currently, accurate detection of phorate mainly relies on analytical techniques such as gas chromatography tandem mass spectrometry (GC-MS) and liquid chromatography tandem mass spectrometry (LC-MS) [11,12,13,14]. Even though these methods have shown sensitivity and reliability for phorate detection, the requirement of sophisticated instruments and skillful technicians to perform the pre-treatment process and instrumental analysis makes them unfit for rapid and on-site screening [15]. To address this issue, some rapid detection methods have been developed for this purpose. For example, various biosensors have been introduced utilizing the enzyme inhibition effect of phorate to acetylcholinesterase (AChE) [16,17]. However, these methods are not suitable for the specific determination of phorate, as other organophosphorus and carbamates pesticides also show AChE inhibition activity. Other efforts have been devoted to developing aptasensors for this task. For instance, Sun and coworkers reported a colorimetric aptasensor using metal organic framework (MOF)-derived materials for the detection of organophosphorus pesticides including phorate [18]. They also introduced electrochemical and electrochemiluminescence aptasensors using hybrid nanocomposites for signal output [19,20]. In another study, Sharma and coworkers described a colorimetric aptasensor for phorate detection [21]. Although these methods can serve as promising platforms for the rapid determination of phorate in agricultural products, the practical applications of aptasensors are still at an early stage.

Comparing with aptamers, antibodies exhibit advantages in terms of chemical stability, binding specificity and affinity for serving as the recognition element [22,23]. Benefiting from these merits, immunochromatography assay (ICA) using antibodies as the recognition element has been in developed and widely applied for rapid and on-site testing of various contaminants in agricultural products, such as pesticide residues, mycotoxins, veterinary drugs and foodborne pathogens [24,25,26,27,28,29]. In China, commercially available test strips have been developed for rapid screening of phorate. However, the commercial kits are based on the traditional colloidal gold-based ICA for signal readout, and the limit of detection (LOD) of the test strip is limited to the level of 0.1 mg/kg. This sensitivity cannot satisfy the requirement of rapid screening and could lead to false-negative results. To overcome this issue, it is necessary to either screen new antibodies to improve the binding affinity or apply new signal readout techniques to improve the sensitivity. In this study, surface-enhanced Raman spectroscopy (SERS) has been introduced to address the signal readout issue for ICA detection of phorate.

SERS is regarded as an advanced analytical technique that shows promising applications in many fields [30]. Since the early reports in the 1970s, a great deal of effort has been devoted to understanding the mechanisms and expanding its applications. The Raman scattering signal of the target molecule can be significantly amplified when it is attached at the surface of noble metal nanoparticles (Au, Ag or Cu). This phenomenon can be explained by the electromagnetic mechanism, which suggests that the localized surface plasmon resonance (LSPR) effect generated by noble metal nanoparticles struck by incident light significantly enhances the Raman scattering signal [31]. Another widely accepted hypothesis is the chemical enhancement mechanism, which emphasizes the charge transfer between the attached molecule and the noble metal surface [32]. In some cases, both mechanisms contribute to the observed SERS signals. As an analytical technique, SERS presents many advantages including ultrahigh sensitivity, rapid detection, a “fingerprint” spectrum, non-interference from the aqueous medium, non-destructive assay and portability of devices [33,34]. SERS can achieve sensitivity at the single-molecule level, making it one of the most sensitive analytical tools to date [35,36]. Benefitting from these merits, it has been applied in various areas such as food safety, point-of-care testing, environmental monitoring and security checks [37,38,39,40]. The combination of SERS and ICA has been proven to effectively improve the sensitivity and capability of quantitative analysis compared with traditional ICA methods [41]. So far, SERS-ICA has been developed for the detection of various targets [42,43], but the sensitive detection of phorate is yet to be reported.

Herein, a sensitive and robust method has been developed for the rapid detection of phorate in vegetables. By combining ICA with SERS, the sensitivity of the assay is remarkably improved compared with the commercially available kits. In order to simplify the pre-treatment process and enhance the signal intensity, a hybrid magnetic nanoprobe was designed and prepared by modifying Fe_3_O_4_ nanobeads with Au nanoseeds. Au reduction reaction was further performed to allow the growth of a Au layer on the magnetic core. The resulting Fe_3_O_4_@Au nanoparticles were functionalized with a Raman tag molecule and phorate antibodies. For phorate detection, the synthesized Fe_3_O_4_@Au@DTNB-Ab probes were mixed with the testing solution. After enriching with a magnet, the probe was loaded on the test strip and SERS signals were recorded. Compared with the traditional colloid gold-based assay, the proposed SERS-ICA method presented clear advantages in both sensitivity and the capability of quantitative analysis.

## 2. Materials and Methods

### 2.1. Reagents and Materials

Ferric chloride hexahydrate (FeCl_3_·6H_2_O, 98%), ethylene glycol (99%), sodium citrate (99%) and chloroauric acid (HAuCl_4_, 99%) were purchased from Energy Chemicals (Shanghai, China). Anhydrous sodium acetate (99%), sodium borohydride (NaBH_4_, 99%), polyethyleneimine (PEI, M. W. 10,000, 99%) and 5,5′-dithiobis (2-nitrobenzoic acid) (DTNB, 98%) were obtained from Macklin Biochemicals (Shanghai, China). Hydroxylamine hydrochloride (NH_2_OH·HCl, 98%), polyvinylpyrrolidone (PVP, M. W. 40,000, 99%), *N*-hydroxysuccinimide (NHS, 99%) and 1-ethyl-3-(3-dimethylaminopropyl)carbodiimide (EDC) were purchased from Aladdin (Shanghai, China). Phorate, acephate, cyromazine, carbofuran, methylparathion, phoxim, malathion and chlorpyrifos reference materials were purchased from ALTA Scientific (Tianjin, China). Bovine serum albumin (BSA, 97%), phosphate-buffered saline (PBS), Tris-HCl, HEPES, MES and PBST buffer solutions were obtained from Solarbio Life Sciences (Beijing, China). Phorate antibody and phorate test strips were provided by Kwinbon Bio (Beijing, China). Ethanol, methanol, and acetonitrile (LCMS grade) were purchased from Fisher Scientific (Waltham, MA, USA).

### 2.2. Instruments

SERS spectra were recorded using a Raman microscope (DXR3, Thermo Fisher Scientific, Waltham, MA, USA) equipped with a 780 nm laser as the excitation light source. Transmission electron microscopy (TEM) images were obtained using a transmission electron microscope (HT-7700, Hitachi, Tokyo, Japan). Ultraviolet–visible (UV–Vis) extinction spectra were recorded using a multimode plate reader (EnVision, Perkin Elmer, Waltham, MA, USA). Ultra-pure water used in the experiments was purified using a water purification system (Milli-Q Direct 8/16, Merck, Darmstadt, Germany). LC-MS analysis of the celery extraction solution was performed using an LC-MS system (Xevo TQ-S, Waters, Milford, MA, USA). Other small instruments used in the experiments included an electronic balance (BSA2238, Sartorius, Göttingen, Germany), zeta potential analyzer (Zetasizer Nano ZS, Malvern, Worcestershire, UK) vacuum oven (DZG-6020, Shanghai SENXIN, Shanghai, China), ultrasonic cleaner (SK2210HP, Shanghai KEDAO, Shanghai, China), magnetic stirrer hot plate (MS-H280, DLAB, Beijing, China), and vortex mixer (Vortex 1, IKA Works, Breisgau, Germany).

### 2.3. Synthesis and Characterization of Fe_3_O_4_@Au Nanoparticles

Magnetic nanoparticles were synthesized by hydrothermal reaction. In detail, 2.70 g of FeCl_3_·6H_2_O and 50 mL of ethylene glycol was added into a beaker and stirred at room temperature for 30 min to obtain a clear yellow solution. Anhydrous sodium acetate (5.75 g) was added and stirred for another 30 min to generate a yellow turbid solution, which was sealed in a 100 mL tetrafluoroethene vessel and heated at 200 °C in an oven for 8 h. The reaction vessel was cooled at room temperature overnight. The black precipitate was collected using a neodymium magnet, washed with ethanol and ultrapure water three times, and then dried in vacuum at 60 °C for 12 h to obtain the synthesized Fe_3_O_4_ nanobeads.

Au nanoseeds were synthesized following the method outlined in the literature [44,45]. In detail, 1.5 mL of 1% sodium citrate solution and 0.5 mL of 24.3 mM HAuCl_4_ solution was added into 50 mL of ultrapure water. The reaction solution was stirred at 700 r/min at room temperature. After 1 min, 0.5 mL of 0.075% NaBH_4_ solution was added dropwise and stirred at 800 r/min for 12 h to generate 3 nm of Au nanoseeds. For the synthesis of 25 nm of Au nanoseeds, 2 mL of 1% HAuCl_4_ solution was added into 100 mL of ultrapure water, stirred, and heated to boil. Then, 1.3 mL of 2% sodium citrate solution was poured in, and the reaction solution turned wine red. It was kept at boiling and stirring for another 10 min, and then cooled to room temperature to obtain 25 nm of Au nanoseeds.

For the preparation of Fe_3_O_4_@Au_25/3_ nanocomposite, 10 mg of Fe_3_O_4_ nanobeads were coated with a layer of PEI by sonicating in 2 mL 5 mg/mL PEI solution for 1 h, enriched using a magnet and washed with ultrapure water five times to obtain Fe_3_O_4_@PEI. After that, 20 nm and 3 nm of Au nanoseeds were assembled on the surface of Fe_3_O_4_. In detail, the prepared Fe_3_O_4_@PEI was mixed with 100 mL of 25 nm Au nanoseed colloid, sonicated for 30 min, and washed with ultrapure water three times to obtain the Fe_3_O_4_@Au_25_ nanocomposite. Then, it was transferred into 50 mL of 3 nm Au nanoseed colloid, sonicated for another 30 min, and washed with ultrapure water three times to obtain the Fe_3_O_4_@Au_25/3_ nanocomposite.

To synthesize the Fe_3_O_4_@Au nanoparticles, the sonochemically assisted growth of Au nanoseeds on the Fe_3_O_4_ surface was carried out following a method outlined in the literature with slight modification [46]. In detail, Fe_3_O_4_@Au_25/3_ was dispersed in 100 mL of 0.5 mmol/L HAuCl_4_ solution, and 1 mL of 100 mg/mL NH_2_OH·HCl solution was rapidly added under sonication at 30 °C. After 5 min, 300 mg of PVP was added. The reaction mixture was sonicated for 10 min, followed by separation of the products using a magnet and washing with ultrapure water three times. The final product was dispersed in 5 mL of ultrapure water to obtain the Fe_3_O_4_@Au solution with a concentration of 2 mg/mL. The dispersion solution was stored at 4 °C for further use.

The morphology and particle size of the synthesized materials at each stage were characterized using TEM. UV–Vis extinction spectra of the synthesized Fe_3_O_4_, Au nanoseeds and Fe_3_O_4_@Au nanoparticles were also recorded using a multimode plate reader. Zeta potentials of the synthesized nanocomposites at each stage were measured using a laser diffraction particle analyzer.

### 2.4. Preparation of Fe_3_O_4_@Au@DTNB-Ab Probe

The synthesized Fe_3_O_4_@Au nanoparticles were modified by adding 1 μmol/L of DTNB for 5 min. The obtained product was resuspended in 1 mL of 100 mmol/L MES buffer. In this buffer solution, EDC and NHS were added in advance, with the final concentration of 10 mmol/L for EDC and 100 mmol/L for NHS. After sonicating for 15 min, the functionalized probes were separated using a magnet, and then 2 mL of phorate antibody solution at different concentrations were added. The mixture was incubated in 4 mL of 10 mmol/L PBST buffer solution (pH 7.4, with 0.05% Tween-20) under vortex oscillation for 10 min. Excessive carboxyl reactive sites were blocked by adding 20 μL of 10% BSA for 1 h. Finally, the Fe_3_O_4_@Au@DTNB-Ab probes were washed twice with PBST buffer and resuspended in 200 μL of PBS buffer solution.

### 2.5. Optimization of the SERS-ICA Conditions

In order to establish the optimal conditions for the SERS-ICA detection of phorate, several key parameters were optimized in this study, including the concentration of the coating antibodies and the composition and pH of the running buffer. Moreover, the type and proportion of organic solvent in the incubation buffer were also examined due to the poor solubility of phorate in aqueous medium. For these optimization studies, 100 ng/mL of phorate standard solution was used as the analyte, and the inhibition rate was employed to evaluate the performance of the SERS-ICA under each condition. The inhibition rate was calculated following the literature method:

$$\mathrm{Inhibition}\;\mathrm{rate}=\left(\frac{T_{0}}{C_{0}}-\frac{T_{1}}{C_{1}}\right)/\frac{T_{0}}{C_{0}}\times 100\%$$

*T*_0_ and *C*_0_ represent the SERS signal intensity on the T line and C line of the blank solution, while *T*_1_ and *C*_1_ correspond to the SERS signal intensity on the T line and C line with 100 ng/mL of phorate as the analyte.

### 2.6. Quantitative Analysis of Phorate Using SERS-ICA

For detecting phorate on the test strip, the prepared Fe_3_O_4_@Au@DTNB-Ab probes were mixed with phorate standard solutions with different concentrations (1, 5, 10, 20, 50, 100, 200 and 1000 ng/mL, respectively) at a ratio of 1:1. The mixture was incubated at 37 °C for 10 min. Then, the probes were enriched using a magnet. The supernatant was removed, and the probes were resuspended in HEPES buffer. For the SERS-ICA test, 100 μL of final solution was pipetted on the sample pad of the test strip. After 5 min, color changes were observed on the test line (T line) and control line (C line) of the nitrocellulose (NC) membrane. SERS spectra on the T line and C line were recorded by placing the test strip under a Raman microscope (see Supplementary Information, Figure S1). For each line, three measurements were conducted on different spots, and the averaged signal was calculated as the final SERS intensity.

### 2.7. Analysis of Spiking Samples

Celery purchased from the local market was washed with tap water and cut into small pieces of approximately 1 cm. Samples weighing 5 g (with an accuracy of ±0.1 g) were weighted in 50 mL centrifuge tubes, then 10 mL of acetonitrile was pipetted in, and the centrifuge tubes were placed in a shaker. After shaking for 5 min, 5 mL of supernatant was taken and dried with nitrogen gas. The remaining residue was redissolved in 1 mL of HEPES buffer with 20% methanol.

For the preparation of spiked samples, the extraction solutions were first analyzed using the LC-MS method, to make sure no phorate was present in the blank samples. After that, phorate stock solutions with different concentrations were added to the solution. The final concentrations of phorate were set at 10, 20 and 40 ng/mL, respectively. The SERS-ICA detection of the prepared samples was carried out following the same procedure as that in Section 2.6.

In the current study, SERS spectra were recorded with specific parameters: laser power: 5 mW; excitation wavelength: 780 nm; acquisition time: 5 s; objective lens: 10×; scanning range: 700~1800 cm^−1^; fluorescence correction factor: 5. Origin 2018 (OriginLab, Northampton, MA, USA) was employed for the analysis of recorded spectra, including baseline correction and linear fitting.

## 3. Results and Discussion

### 3.1. Principle of SERS-ICA for Phorate Detection

**Figure 1:**
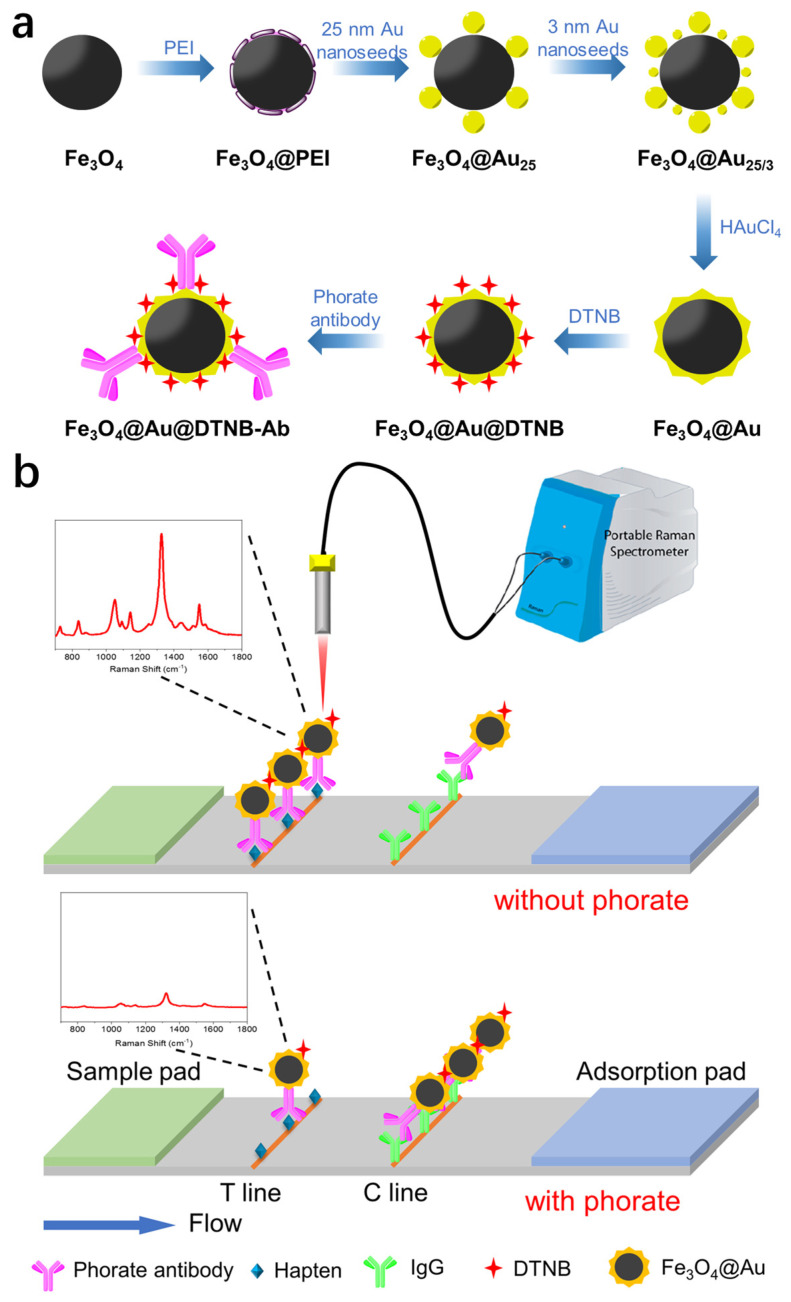
(**a**) Schematic illustration of the preparation of the Fe_3_O_4_@Au@DTNB-Ab probe. (**b**) Schematic illustration of the SERS-ICA method for detecting phorate.

The preparation process of the proposed Fe_3_O_4_@Au@DTNB-Ab probe is illustrated in Figure 1a. Fe_3_O_4_ magnetic nanobeads were first synthesized through hydrothermal reaction. Positively charged polymer was coated on the Fe_3_O_4_ nanobead, followed by the assembly of Au nanoseeds of different sizes. After introducing Au precursor and reductant, a rough layer of Au could grow on the Au nanoseeds. The prepared Fe_3_O_4_@Au nanoparticles can serve as a SERS-active substrate, as well as performing fast enrichment during the sample pre-treatment process. After functionalizing the Fe_3_O_4_@Au nanoparticles with DTNB and phorate antibodies, the final product was obtained as Fe_3_O_4_@Au@DTNB-Ab probes for SERS-ICA. DTNB was chosen as the Raman tag molecule due to its high and stable SERS activity, as well characteristic SERS spectrum, which is critical for avoiding interference and realizing high sensitivity.

The SERS-ICA detection of phorate was carried out by mixing the prepared Fe_3_O_4_@Au@DTNB-Ab probe with the testing solution. The mixture solution was incubated at 37 °C, allowing the binding between the target molecules and the antibodies on the outer layer of the probes. After magnetic enrichment, the incubation buffer was replaced by the running buffer, and the enriched solution was loaded on the sample pad of the test strip. During the chromatography process, the probe in the mixed solution successively passed through the T line and C line of the NC membrane. As illustrated in Figure 1b, in the absence of the target phorate, the antibodies on the outer layer of the probes bind with the phorate-BSA hapten anchored on the T line, while the excess probes migrate to the C line and are captured by the goat anti-mouse IgG. SERS signals on both T line and C line were measured, and the ratio of SERS intensity on the T line and C line (T/C ratio) could be calculated. Upon the addition of phorate in the testing solution, the antibodies on the Fe_3_O_4_@Au@DTNB-Ab probe bind will with the target molecules first. After enriching the probes, the resuspended solution was applied on the test strip following the same procedure. Because the antibodies on the probes were pre-occupied by the phorate molecules, they could not conjugate with the hapten, resulting in a reduced SERS signal on the T line. Phorate can be qualitatively detected based on the change in T/C ratio. Moreover, quantitative detection can also be realized by establishing the relationship between different concentrations of phorate and the corresponding T/C ratios. Compared with the traditional colloidal gold-based immunochromatographic strip, SERS-ICA allows quantitative detection of the target molecule with a much higher sensitivity, as the SERS signal readout provides more sensitive measurement compared with visual test.

### 3.2. Preparation of the Fe_3_O_4_@Au@DTNB-Ab Probe

**Figure 2:**
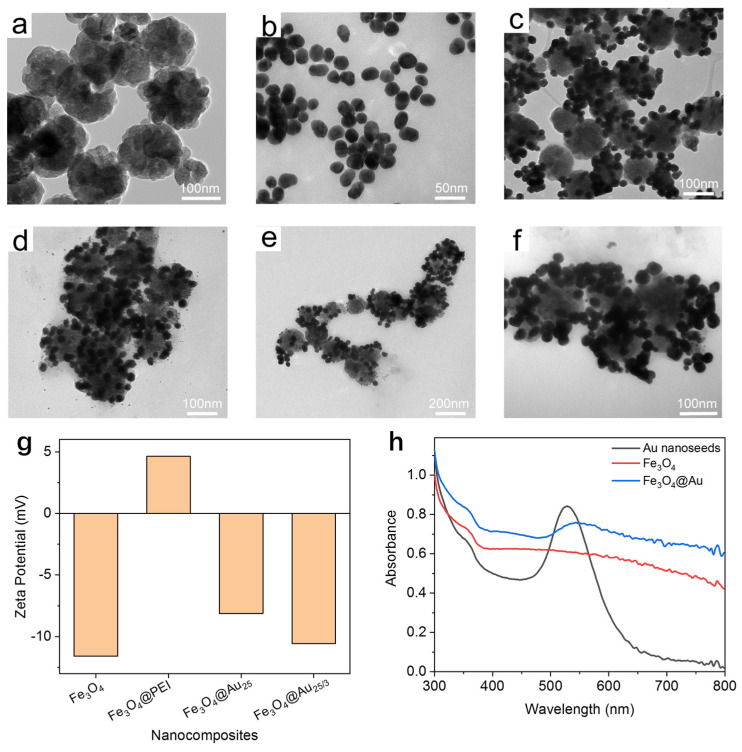
Characterization of the synthesized nanomaterials. (**a**–**f**) TEM image of the synthesized Fe_3_O_4_ nanobeads (**a**), 25 nm Au nanoseeds (**b**), Fe_3_O_4_@Au_25_ nanocomposites (**c**), Fe_3_O_4_@Au_25/3_ nanocomposites, (**d**) and Fe_3_O_4_@Au nanoparticles (**e**,**f**). (**g**) Zeta potential measurements of the synthesized nanocomposites at different stages of preparation. (**h**) UV–Vis extinction spectrum of the synthesized Fe_3_O_4_ nanobeads (red), Au nanoseeds (black), and Fe_3_O_4_@Au nanoparticle (blue), respectively.

As presented in Figure 2a, the synthesized Fe_3_O_4_ nanobeads exhibited a relatively even particle size distribution, with an average diameter of 130 nm. Small-sized Fe_3_O_4_ was chosen as the core to control the size of the final Fe_3_O_4_@Au@DTNB-Ab probe, thus facilitating the chromatography process on the NC membrane. Au nanoseeds of two sizes were synthesized by using different reductants. The size of the larger Au nanoseeds was verified to be 25 nm in the TEM image (see Figure 2b). After that, the Fe_3_O_4_ nanobeads were encapsulated with a layer of PEI to make the surface positively charged. This can be verified by measuring the zeta potential of the nanobeads. As presented in Figure 2g, the zeta potential of Fe_3_O_4_ changed from −11.59 mV to 4.63 mV after coating with PEI. Through electrostatic interaction, negatively charged Au nanoseeds were adsorbed on the outside of the Fe_3_O_4_. As shown in Figure 2c,d, Au nanoseeds were densely coated on the Fe_3_O_4_ core. And the average size of the Fe_3_O_4_@Au_25_ nanocomposite increased to 163 nm. The 3 nm Au nanoseeds were then introduced to facilitate the growth of the Au layer. The average size of the nanocomposite was almost unchanged after decorating with 3 nm Au nanoseeds. Figure 2e,f presents the Fe_3_O_4_@Au nanoparticles after reducing HAuCl_4_ on the outer layer of the nanocomposite. The average diameter of the Fe_3_O_4_@Au nanoparticles was approximately 183 nm, according to the TEM images. Nanoparticles of this size can easily migrate on the NC membrane of the test strip. The roughened surface of the synthesized Fe_3_O_4_@Au nanoparticles could provide strong SERS enhancement for the DTNB tag molecule, which is crucial for guaranteeing the sensitivity of the SERS-ICA measurement. The zeta potentials of the prepared nanocomposites at different steps are also presented in Figure 2g. Apart from PEI functionalization, the nanocomposites were negatively charged during the synthesis. In Figure 2h, UV–Vis extinction spectra of Fe_3_O_4_ nanobeads, Au nanoseeds and Fe_3_O_4_@Au nanoparticles are presented. Maximum adsorption at 528 nm was observed for 25 nm Au nanoseeds, while a broad adsorption peak at 542 nm appeared for Fe_3_O_4_@Au nanoparticles, indicating successful coating of Au on the surface. Apart from TEM images and UV–Vis spectra, element mapping images and energy dispersive spectroscopy (EDS) were also acquired to verify the chemical composition of the synthesized Fe_3_O_4_@Au nanoparticles (see Supplementary Information, Figure S2).

**Figure 3:**
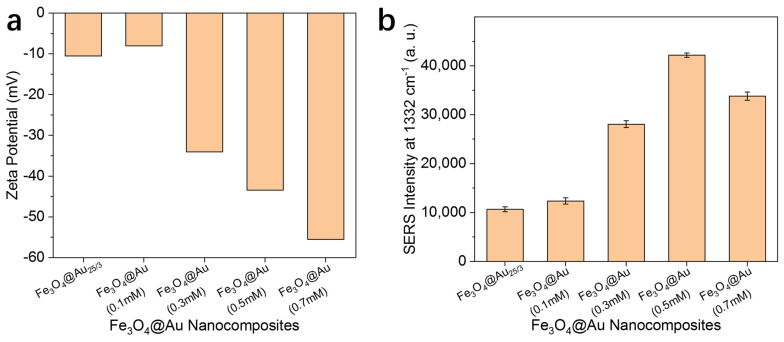
Optimization of the Fe_3_O_4_@Au nanoparticles. Zeta potential measurements (**a**) and SERS intensity at 1332 cm^−1^ (**b**) of the synthesized Fe_3_O_4_@Au nanoparticles with different HAuCl_4_ concentrations.

In order to achieve ideal SERS activity, the preparation of Fe_3_O_4_@Au nanoparticles was optimized by adjusting the concentration of Au precursor during the Au growth process. Four concentrations of HAuCl_4_ were examined (0.1, 0.3, 0.5 and 0.7 mmol/L). As presented in Figure 3a, the zeta potentials of the synthesized Fe_3_O_4_@Au gradually decreased with the increase in HAuCl_4_ concentration, suggesting that a more negatively charged Au surface was formed during the growth process. The SERS activity of the prepared Fe_3_O_4_@Au nanoparticles was further studied by recording SERS spectra of DTNB standard solution. Judging by the result presented in Figure 3b, the concentration of HAuCl_4_ has a clear impact on the SERS activity of the Fe_3_O_4_@Au nanoparticles, presumably due to the roughness of the Au outer layer. When the concentration of the Au precursor was adjusted to 0.5 mmol/L, the synthesized Fe_3_O_4_@Au exhibited the maximum SERS activity, and this condition was used in subsequent studies.

### 3.3. Optimization of SERS-ICA Conditions

**Figure 4:**
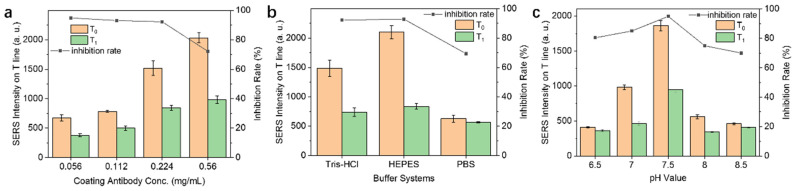
Optimization of coating antibody concentration, buffer solution, and pH. SERS intensity at 1332 cm^−1^ and inhibition rates with different coating antibody concentrations (**a**); different running buffer solutions (**b**) and pH values (**c**). T_1_ and T_0_ represent the SERS intensity of the T line with and without phorate, respectively.

After optimizing the Fe_3_O_4_@Au nanoparticles, they were functionalized with DTNB and phorate antibodies through the DEC/NHS coupling reaction. The antibody serves as the recognition element for phorate detection. Therefore, the distribution of phorate antibodies on the nanocomposite is a key factor for the performance of the SERS-ICA assay. In order to optimize the modification process, the concentration of phorate antibodies was evaluated in this experiment. Four different concentrations of phorate antibody solutions were prepared from 0.056 to 0.560 mg/mL. The result is presented in Figure 4a. The performance of the assay was evaluated by calculating the inhibition rate following the equation described in the Section 2. A higher inhibition rate indicates better performance for phorate detection. In addition to the inhibition rate, SERS intensities on the T line with and without phorate (assigned as T_1_ and T_0_, respectively) are also considered as important indicators for evaluating SERS-ICA performance. As shown in Figure 4a, the concentration of antibody solution has a strong effect on the SERS intensity measured on the T line. In general, the intensity of SERS signal gradually enhanced upon the increase in antibody concentration. Judging by the inhibition rates under different conditions, the inhibition rate clearly decreased when the concentration of antibodies was higher than 0.224 mg/mL. Considering the outcomes of both the SERS intensity and inhibition rate, the optimal concentration of phorate antibodies was determined to be 0.224 mg/mL, and it was used in subsequent studies.

To facilitate the chromatography process, the composition of the running buffer is another key factor. An improper buffer system and pH could cause the detachment of hapten and antibodies from the NC membrane, resulting in a diminished signal readout. In this experiment, three types of commonly used buffer solutions were examined, including Tris-HCl, HEPES, and PBS buffer. As illustrated in Figure 4b, HEPES buffer produced the highest SERS intensity and inhibition rate, while PBS buffer is unsuitable in the current system. As a result, HEPES buffer was chosen as the running buffer in this study. Other than the composition of buffer solution, the pH value of the buffer system is also important. Optimal pH would provide an ideal environment for antigen–antibody interaction. In this experiment, five pH conditions between 6.5 and 8.5 were tested, and the results are presented in Figure 4c. Under pH 7.5, the SERS-ICA produced the highest inhibition rate, and the SERS intensity on the T line was also at the optimal level. Therefore, the pH of the running buffer was adjusted to 7.5 and applied in the following experiments.

**Figure 5:**
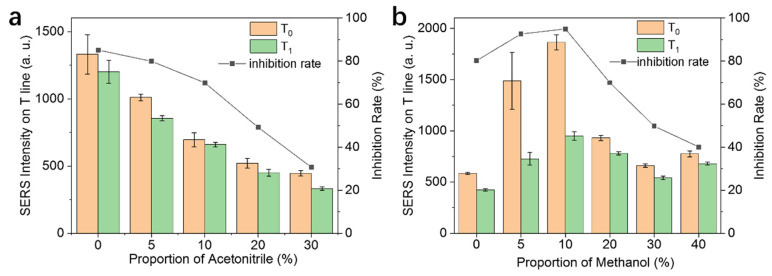
Optimization of organic solvents in the incubation buffer system. SERS intensity and inhibition rate with different proportions of acetonitrile (**a**) and methanol (**b**) in the incubation buffer system.

Due to the poor water-solubility of phorate, it is necessary to introduce a small amount of organic solvent to ensure that the target molecules are fully dissolved during the incubation process. However, the type and proportion of the additional solvent need to be investigated. An excess amount of organic solvent could result in the dysfunction of antibodies and thus affect the antigen–antibody interaction. To guarantee the solubility of phorate and minimize the interference of organic solvent, two types of solvents (acetonitrile and methanol) were evaluated with different proportions in the incubation buffer. As illustrated in Figure 5, the presence of acetonitrile had a clear effect on the SERS intensity and inhibition rates of the SERS-ICA. Presumably, this might be caused by the denaturation of antibodies on the probes, as well as the destabilization of DTNB tag molecules on the Fe_3_O_4_@Au@DTNB-Ab probes. In contrast, the use of a low ratio of methanol led to improvements in the SERS intensity and inhibition rate, as it may facilitate the dissolving of phorate in the incubation buffer. When the proportion of methanol was higher than 10%, clear decreases in the SERS intensity and inhibition rate were observed. Thus, the optimal condition for organic solvent is set as 10% methanol for the incubation process.

### 3.4. Signal Uniformity, Reproducibility and Specificity of the SERS-ICA for Phorate Detection

**Figure 6:**
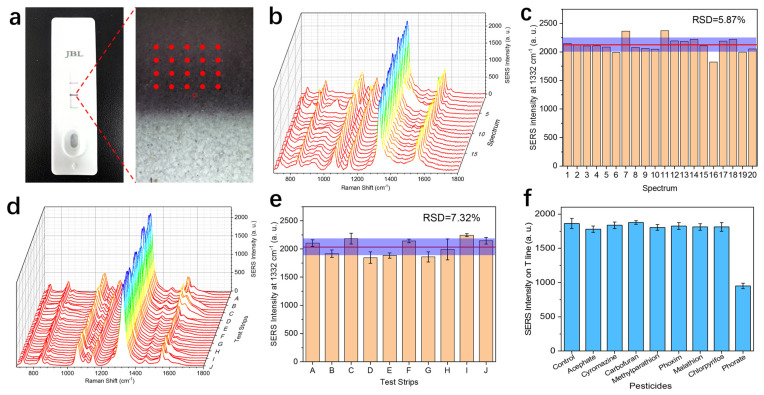
Evaluation of signal uniformity, reproducibility, and specificity of the SERS-ICA method for phorate detection. (**a**) Optical image of the test strip after loading the Fe_3_O_4_@Au@DTNB-Ab probe without phorate (left), and the microscopic image of the T line under a 10× objective lens. (**b**) SERS spectra recorded from 20 spots on the T line labeled in the microscopic image. (**c**) SERS intensity at 1332 cm^−1^ of the 20 spectra presented in (**b**). The red line represents the average intensity of the 20 spectra, and the intensity variation is marked with the purple bar in the graph. (**d**) SERS spectra recorded from the T lines of 10 different test strips (A to J). For each test strip, 3 spectra were taken on different spots. (**e**) SERS intensity at 1332 cm^−1^ of the 30 spectra presented in (**d**). (**f**) SERS intensity at 1332 cm^−1^ of the T line for phorate (10 ng/mL) versus other pesticides: acephate, cyromazine, carbofuran, methylparathion, phoxim, malathion, and chlorpyrifos (100 ng/mL).

In this experiment, the prepared Fe_3_O_4_@Au@DTNB-Ab probes were loaded on the sample pad without the addition of phorate. As shown in Figure 6a, after migrating on the NC membrane, a dark line can be clearly observed on the T line, indicating that the conjugation between the probe and phorate-BSA hapten occurred. Using the Raman microscope system, it is convenient to identify the point of interest and record the corresponding SERS spectrum. To evaluate the SERS signal uniformity of the SERS-ICA method, 20 spots on the T line were picked in a rectangular matrix form. Area scanning was carried out in an area of 300 μm^2^. The recorded spectra are presented in Figure 6b, and the SERS intensity at 1332 cm^−1^ is summarized in Figure 6c. As presented, the signal uniformity on the T line is relatively good, with a calculated relative standard deviation (RSD) of 5.87%, which is comparable to previous research [47]. Benefiting from the relatively small size and good dispersity of the probes, they were evenly adsorbed on the T line and produced desirable signal uniformity, which is essential for accurate and repeatable assay.

After verifying the signal uniformity on a single test strip, it is necessary to further investigate the reproducibility of the SERS signal from different batches of test strips. For this purpose, 10 test strips from different batches purchased from Kwinbon Bio were chosen. The prepared Fe_3_O_4_@Au@DTNB-Ab probes were loaded on the test strips and migrated under the same conditions. For each test strip, three spots were randomly selected on the T line, and the SERS spectra were recorded. As shown in Figure 6d,e, the SERS intensities at 1332 cm^−1^ are relatively consistent among the tested strips, with a calculated RSD of 7.32%. This result further demonstrates the reproducibility of the SERS-ICA method between different batches of commercial test strips. Moreover, in light of the stability of the test strips, no displacement of the hapten and antibody on either the T line or C line was observed under the optimized chromatographic conditions, which is also vital for achieving consistent and reliable assay.

To examine the specificity of the established SERS-ICA method for phorate detection, a series of commonly used organophosphorus and carbamates pesticides were selected as the potential interferences, including acephate, cyromazine, carbofuran, methyl parathion, phoxim, malathion, and chlorpyrifos. The chemical structures of the tested pesticides were drawn in the Supplementary Information (see Figure S3). Some of the selected pesticides have the same motif as phorate, while other pesticides are often simultaneously detected in vegetables [48]. As presented in Figure 6f, the concentration of the seven interreference pesticides is 10 times higher than the concentration of phorate. Judging by the SERS intensity at 1332 cm^−1^ on the T line, among the tested eight pesticides, only phorate showed a clear inhibition effect, suggesting the desirable selectivity of the SERS-ICA method. This good selectivity can be attributed to the specificity of the phorate antibody, which did not cross-react with other interferences.

### 3.5. Sensitivity and Linear Range of the SERS-ICA Method

**Figure 7:**
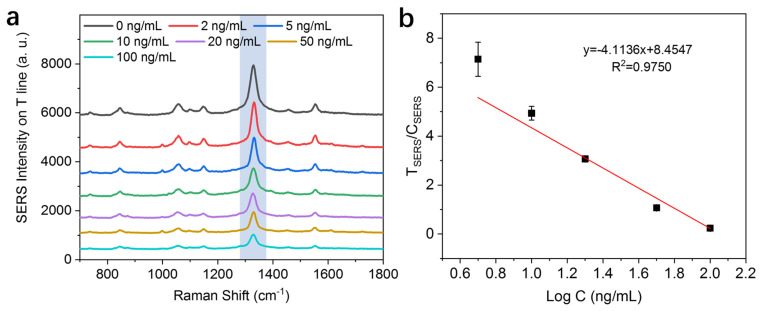
Quantitative detection of phorate using the SERS-ICA method. (**a**) SERS spectra of different concentrations of phorate recorded on the T line of the test strips. The SERS intensity is taken according to the main peak at 1332 cm^−1^, which is labeled by the light blue bar in the graph. (**b**) Linear calibration curves of the ratio of SERS intensity of the T line and C line versus the logarithmic concentrations of phorate.

The results of SERS-ICA detection of different concentrations of phorate are presented in Figure 7. As the concentration of phorate increased, the SERS intensity on the T line decreased accordingly. This was caused by the competitive binding of antibodies from the phorate in the testing solution. The probes conjugated on the T line were reduced as less antibodies were available for binding with hapten, resulting in a weakened SERS signal. By plotting the ratio of SERS intensity of the T line and C line against the logarithm of phorate concentrations, a linear relationship can be established at 5~100 ng/mL, indicating the feasibility of quantitative analysis for phorate. The calibration equation was established as y = −4.1136x + 8.4547, with a correlation coefficient (R^2^) of 0.975. The LOD of this method was calculated as 1 ng/mL, which is significantly lower than that of commercial kits with colloidal gold-based ICA (LOD = 100 ng/mL, according to Kwinbon Bio). It can also satisfy the requirement of MRL set by the Chinese government (0.01 mg/kg). In addition, the magnetic enrichment process can clearly simplify the pre-treatment procedure, making it an attractive approach for the rapid and quantitative detection of phorate.

### 3.6. Spiking Sample Analysis

For the assessment of the performance of SERS-ICA in real samples, celery was selected as the tested sample as the detection of pesticide residues, including phorate, is frequently reported. Blank celery extracting solution was obtained following the procedure described in Section 2.7. After the addition of different volumes of phorate stock solution, the spiked solutions were obtained with final concentrations of 10, 20 and 40 ng/mL, respectively. The spiked samples were analyzed using the established SERS-ICA method. The detected concentrations were calculated according to the previously established calibration equation. As summarized in Table 1, the recovery rates of the spiked sample tests were calculated as 96.7~105.1%, with RSDs between 7.4 and 8.7%. This result indicates that the established SERS-ICA platform can be employed for quantitative detection of phorate residues in celery samples, with high sensitivity and satisfactory accuracy.

## 4. Conclusions

In this study, a SERS-ICA method was described for the rapid and sensitive detection of phorate residue in vegetables. ICA strips are currently widely used for fast and on-site inspection of pesticide residues. However, with the traditional colloidal gold-based ICA platform, the low sensitivity for phorate testing makes it difficult to meet real-world demands. To address this issue, a Fe_3_O_4_@Au@DTNB-Ab probe was prepared by modifying Fe_3_O_4_ magnetic nanobeads with Au nanoseeds, followed by growing the Au nanoseeds to obtain a roughened Au outer layer. After optimizing the Au growth process and the ICA parameters, quantitative detection of phorate was achieved with an LOD of 1 ng/mL and a linear range between 5 and 100 ng/mL. The spiking experiment further demonstrated the performance of this method for detecting phorate in celery extraction solution. By updating the pre-treatment process and signal output technique, the sensitivity of ICA detection of phorate was improved by two orders of magnitude. This study demonstrates that the SERS-ICA method enables rapid and sensitive detection of target molecules without changing the recognition elements. It shows promising future applications for the detection of various pesticides in agricultural products.

## Figures and Tables

**Table 1:** Detection of phorate spiked in celery extraction solutions using the proposed SERS-ICA method.

Added (ng/mL)	Detected by SERS-ICA (ng/mL)	Recovery (%)	RSD (%)
10	10.509	105.1	8.7
20	19.344	96.7	7.4
40	38.912	97.3	8.2

## Data Availability

Data are contained within the article.

## Supplementary Materials

The following supporting information can be downloaded at https://www.mdpi.com/article/10.3390/nano14121046/s1: Figure S1: SERS spectra on the T line and C line of the test strip recorded using a Raman microscope. Figure S2: TEM and elemental mapping images of the synthesized Fe_3_O_4_@Au nanoparticles. (a–d) TEM image (a); elemental mapping images of Au (b), Fe (c), and O (d). (e) Energy dispersive spectroscopy of the synthesized Fe_3_O_4_@Au nanoparticles. Figure S3: Chemical structures of the eight tested pesticides for evaluating the specificity of the SERS-ICA method.
