# Supplementary material for: Preparation of Hybrid Magnetic Nanoparticles for Sensitive and Rapid Detection of Phorate Residue in Celery Using SERS Immunochromatography Assay
Source: Nanomaterials (Basel). 2024 Jun 18;14(12):1046. doi: 10.3390/nano14121046 (PMC11206780; doi:10.3390/nano14121046)
Supplement: Supplementary file 1 [file nanomaterials-14-01046-s001.zip › nanomaterials-3051022-supplementary.pdf]

Preparation of Hybrid Magnetic Nanoparticles for Sensitive and Rapid Detection of Phorate Residue in Celery using SERS Im-munochromatography Assay

(Supplementary Materials)

Xiangyang Li [1], Hean Qian [1,2], Jin Tao [2], Mingshuo Cao [2], Meng Wang [2,*], Wenlei Zhai [2,*]

1 Beijing Laboratory of Food Quality and Safety, Beijing Key Laboratory of Detection and Control of Spoilage Organisms and Pesticide Residue in Agricultural Product, College of Food Science and Engineering, Beijing University of Agriculture, Beijing 102206, China

2 Institute of Quality Standard and Testing Technology, Beijing Academy of Agriculture and Forestry Science, Beijing 100097, China

* Correspondence: wangm@iqstt.cn; zhaiwl@iqstt.cn

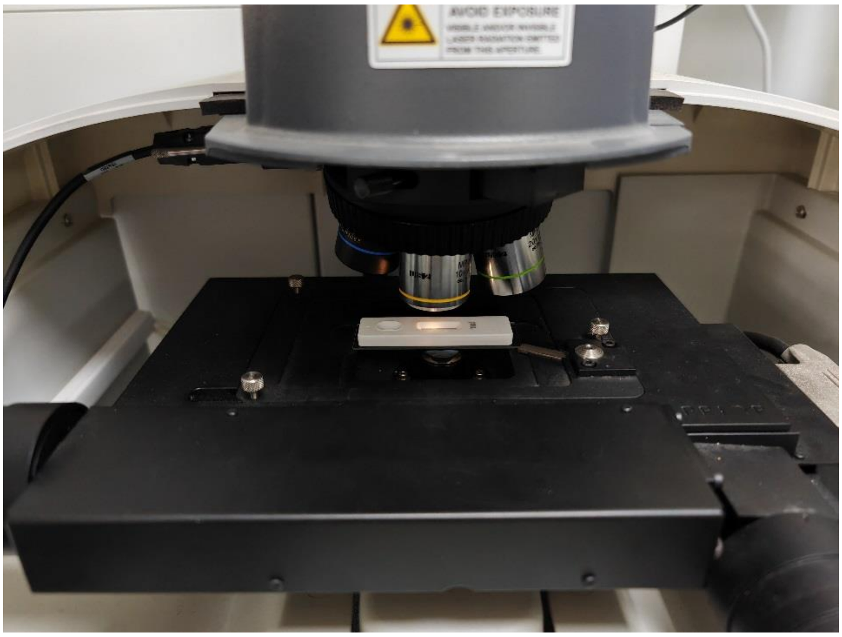

Figure S1. SERS spectra on the T line and C line of the test strip recorded using a Raman microscope

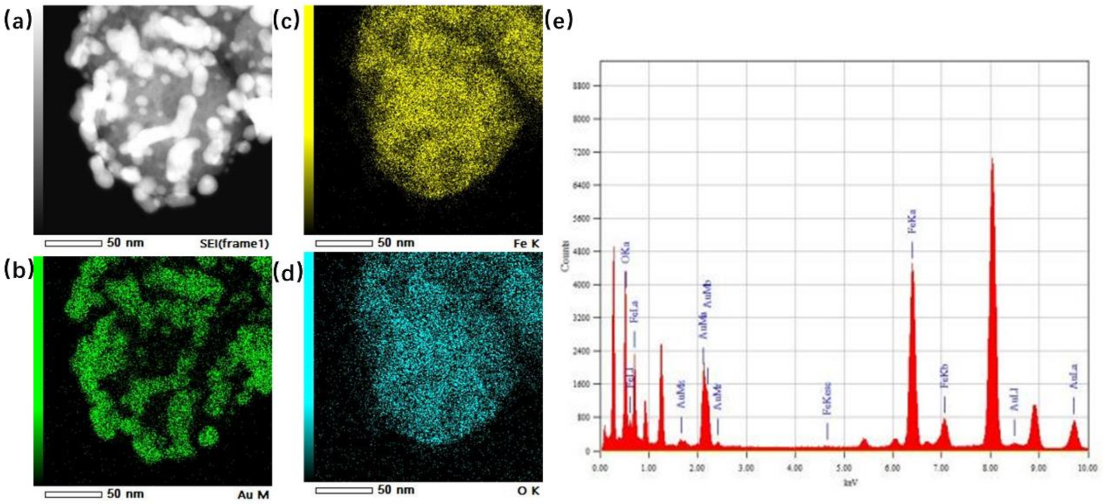

Figure S2. TEM and elemental mapping images of the synthesized Fe_3O_4@Au nanoparticles. (a~d) TEM image (a); elemental mapping images of Au (b); Fe (c) and O (d). (e) Energy dispersive spectroscopy of the synthesized Fe_3O_4@Au nanoparticles.

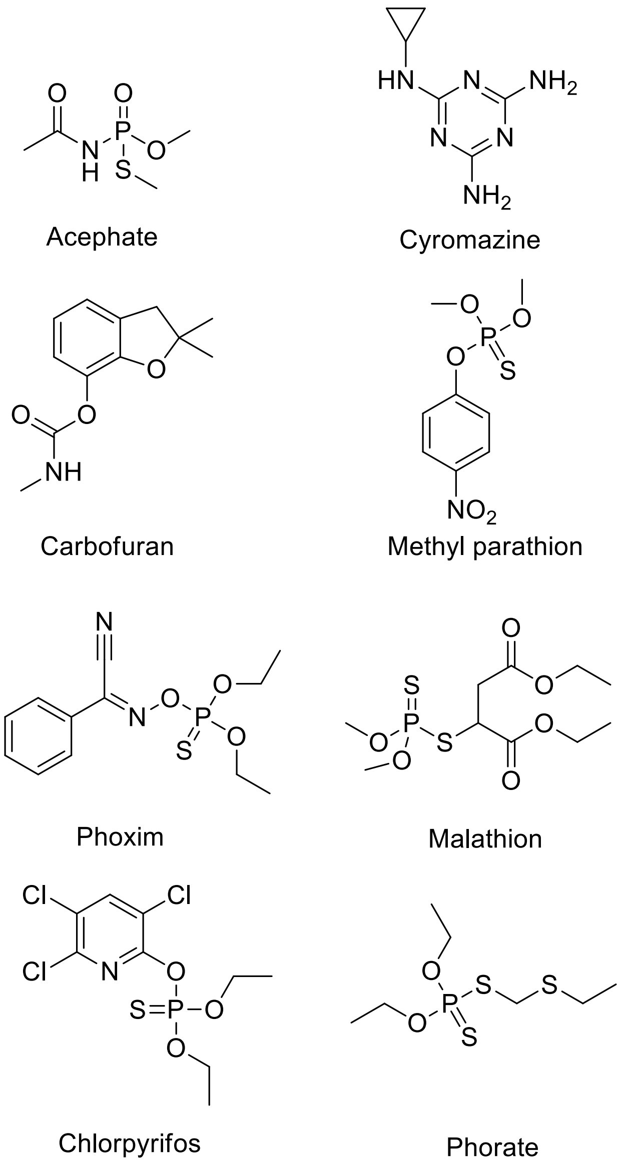

Figure S3. Chemical structures of the eight tested pesticides for evaluating the specificity of the SERS-ICA method.
